# Severe volcanic SO_2_ exposure and respiratory morbidity in the Icelandic population – a register study

**DOI:** 10.1186/s12940-021-00698-y

**Published:** 2021-02-27

**Authors:** Hanne Krage Carlsen, Unnur Valdimarsdóttir, Haraldur Briem, Francesca Dominici, Ragnhildur Gudrun Finnbjornsdottir, Thorsteinn Jóhannsson, Thor Aspelund, Thorarinn Gislason, Thorolfur Gudnason

**Affiliations:** 1 Centre of Public Health Sciences, University of Iceland, Sturlugata 8, Reykjavík, 102 Iceland; 2 Environment and Natural resources, University of Iceland, Sturlugata 7, University of Iceland, 102 Reykjavík, Iceland; 3 Section of Occupational and Environmental Medicine, School of Public Health and Community Medicine, Institute of Medicine, University of Gothenburg, Medicinaregatan 16A, 40530 Gothenburg, Sweden; 4 Department of Epidemiology, Harvard T.H. Chan School of Public Health, 677 Huntington Ave, Boston, MA 02115 USA; 5 Department of Medical Epidemiology and Biostatistics, Karolinska Institutet, Nobels väg 6, SE-171 77 Stockholm, Sweden; 6 Chief Epidemiologist, Directorate of Health, Centre for Health Threats and Communicable Diseases, Barónsstigur 57, 101 Reykjavík, Iceland; 7 The Environment Agency of Iceland, Suðurlandsbraut 24, 108 Reykjavík, Iceland; 8 School of Health Sciences, University of Iceland, Sæmundargata 2, 101 Reykjavík, Iceland; 9 Landspitali – the National University Hospital, Fossvogur, 108 Reykjavík, Iceland; 10 Faculty of medicine, University of Iceland, Vatnsmýrarvegi 16, 101 Reykjavík, Iceland

**Keywords:** Volcanic eruption, Atmospheric transport, Respiratory disease, Epidemiology, Public health

## Abstract

**Background:**

The Holuhraun volcanic eruption September 2014 to February 2015 emitted large amounts of sulfur dioxide (SO_2_). The aim of this study was to determine the association between volcanic SO_2_ gases on general population respiratory health some 250 km from the eruption site, in the Icelandic capital area.

**Methods:**

Respiratory health outcomes were: asthma medication dispensing (AMD) from the Icelandic Medicines Register, medical doctor consultations in primary care (PCMD) and hospital emergency department visits (HED) in Reykjavík (population: 215000) for respiratory disease from 1 January 2010 to 31 December 2014. The associations between daily counts of health events and daily mean SO_2_ concentration and high SO_2_ levels (24-h mean SO_2_ > 125 μg/m3) were analysed using generalized additive models.

**Results:**

After the eruption began, AMD was higher than before (129.4 vs. 158.4 individuals per day, *p* < 0.05). For PCMD and HED, there were no significant differences between the number of daily events before and after the eruption (142.2 vs 144.8 and 18.3 vs 17.5, respectively). In regression analysis adjusted for other pollutants, SO_2_ was associated with estimated increases in AMD by 0.99% (95% CI 0.39–1.58%) per 10 μg/m^3^ at lag 0–2, in PCMD for respiratory causes 1.26% (95% CI 0.72–1.80%) per 10 μg/m^3^ SO_2_ at lag 0–2, and in HED by 1.02% (95% CI 0.02–2.03%) per 10 μg/m^3^ SO_2_ at lag 0–2. For days over the health limit, the estimated increases were 10.9% (95% CI 2.1–19.6%), 17.2% (95% CI 10.0–24.4%) for AMD and PCMD. Dispensing of short-acting medication increased significantly by 1.09% (95% CI 0.49–1.70%), and PCMD for respiratory infections and asthma and COPD diagnoses and increased significantly by 1.12% (95% CI 0.54–1.71%) and 2.08% (1.13–3.04%).

**Conclusion:**

High levels of volcanic SO_2_ are associated with increases in dispensing of AMD, and health care utilization in primary and tertiary care. Individuals with prevalent respiratory disease may be particularly susceptible.

**Supplementary Information:**

The online version contains supplementary material available at 10.1186/s12940-021-00698-y.

## Introduction

SO_2_ (Sulphur dioxide) exposure is associated with respiratory health morbidity and mortality [1, 2] and at higher concentrations (a 10 min mean over 500 μg SO_2_ per m^3^) it is associated with irritation of the respiratory tract in susceptible individuals [2] and can trigger respiratory symptoms such as acute bronchial asthma, pulmonary oedema, and respiratory distress [3] – especially in individuals with hyper-reactivity syndrome [4]. Populations within 100 km of a volcanic eruption have traditionally been considered at risk [5] although studies have found possible health effects of volcanic ash at greater distances [6].

During the Holuhraun volcanic eruption in the Barðarbunga central volcanic system SO_2_ was dispersed widely over Iceland according to meteorological conditions [7], reaching the capital area some 250 km from the eruption site where the 24-h air quality guideline limit for SO_2_, 125 μg/m^3^ [2], was exceeded repeatedly during the fall of 2014. The Barðarbunga volcanic system is located in the central highlands of Iceland which is uninhabited and no humans live within 50 km of the eruption site so although there was no immediate danger, several steps were taken to inform and advise the public of the situation with the dispersed SO_2_ gas. Press briefings and community meetings were held from mid-September and the civil protection agency issued warnings by text messages to all cell phones in the affected areas when high levels of SO_2_ were expected. During the eruption, there were no formal disruptions to daily life such as school closings [8].

The eruption in the fall and winter of 2014–2015 was the largest eruption in Iceland since the Laki eruption in 1783–1784. Some 12 million tons of sulphur dioxide, SO_2_, was emitted from the eruption and the resulting lava field [9], A clinical study of professionals working at the eruption site with very high exposures found no serious health effects associated with exposure, perhaps because they were wearing protective equipment, most importantly, masks [10].

Exposure to SO_2_ from active volcanoes is associated with increased rates of chronic cough and phlegm, as well dry and sore throat [11–18]. although few fatalities have been reported from very high SO_2_ exposure near active volcanoes [19]. In Hawai’i, PM_2.5_ (particle matter with an aerodynamic diameter < 2.5 μm) with a significant amount of volcanic emissions was associated with increased respiratory admissions [20].

While concentration-response relationships between volcanic SO_2_ and respiratory symptoms, many of the studies´ designs and methods leave them prone to bias. For example, symptoms are often self-reported, or participants were aware of their exposure status [21], whereas other studies suffer from a lack of data [22]. Moreover, most of the existing literature pertains to long-term area-wide exposure whereas SO_2_ exposure in Iceland during the Holuhraun eruption was intermittent with few hours or days of high SO_2_ concentrations followed by periods of low SO_2_ concentrations as wind directions changed [7]. With population-based registers on medicine dispensing and health care utilization as well as vigorous air pollution monitoring in the capital area, Iceland provides an ideal setting for studying population health effects of short-term exposure to SO_2_ from a volcanic eruption.

The objective of this study was to study the acute effects of exposure to SO_2_ concentrations from a volcanic source and SO_2_ concentrations above the air quality guideline value of 24 h mean of 125 μg/m^3^) on respiratory health in the general population and to investigate risk differences in subgroups of the population and susceptible groups.

## Material and methods

The Holuhraun volcanic eruption in North-East central Iceland began 31 August 2014 and ended 27 February 2015. The study period was 1 January 2010–31 December 2014, and the time before the eruption was used a reference period. The Holuhraun eruption persisted until end of February 2015, whereas our study period ends 31 December 2014 due to a change in the database recording of events. However, SO_2_ never exceeded the 24-h air quality guideline limit during January and February 2015, although daily mean concentrations were still higher than before the eruption.

The mean population of Iceland during the study period was 320,000 inhabitants. The capital area, Reykjavík and surrounding municipalities, had 205,282 residents at the beginning of the study period, and 215,965 residents at the end [23]. The analysis was restricted to the capital area (residential postcodes 101–171, 200–225, and 270) where adequate information about SO_2_ exposure was available for the whole study period. The Icelandic health care system is state-centred, mainly publicly funded system with universal coverage [24]. We obtained data on respiratory health and individual data on residence (postcode), age, sex and an anonymous personal identification number from 1)) the National Medicines Register; 2) Primary care centres (that function as first point of contact) and 3) Landspitali, the national university hospital, the country’s centre of clinical excellence [2]. All registers are held by the Icelandic Directorate of Health and extraction is subject to approval from the Icelandic Bioethical Committee. From the National Medicines Register we extracted data on dispensing (pharmacy sales to individuals) of prescription anti-asthma medication (AMD) classified by The World Health Organisation Anatomical Therapeutic Chemical code R03. AMD relieve symptoms of asthma and chronic obstructive pulmonary disease, COPD, and are occasionally prescribed to individuals with respiratory infections. Furthermore, AMD is a proxy for respiratory health in a population [25–29]. From the primary care centers (PCC) and hospital emergency department (HED) databases at the Directorate of Health we extracted data on individuals diagnosed with respiratory illnesses.

**Fig. 1:**
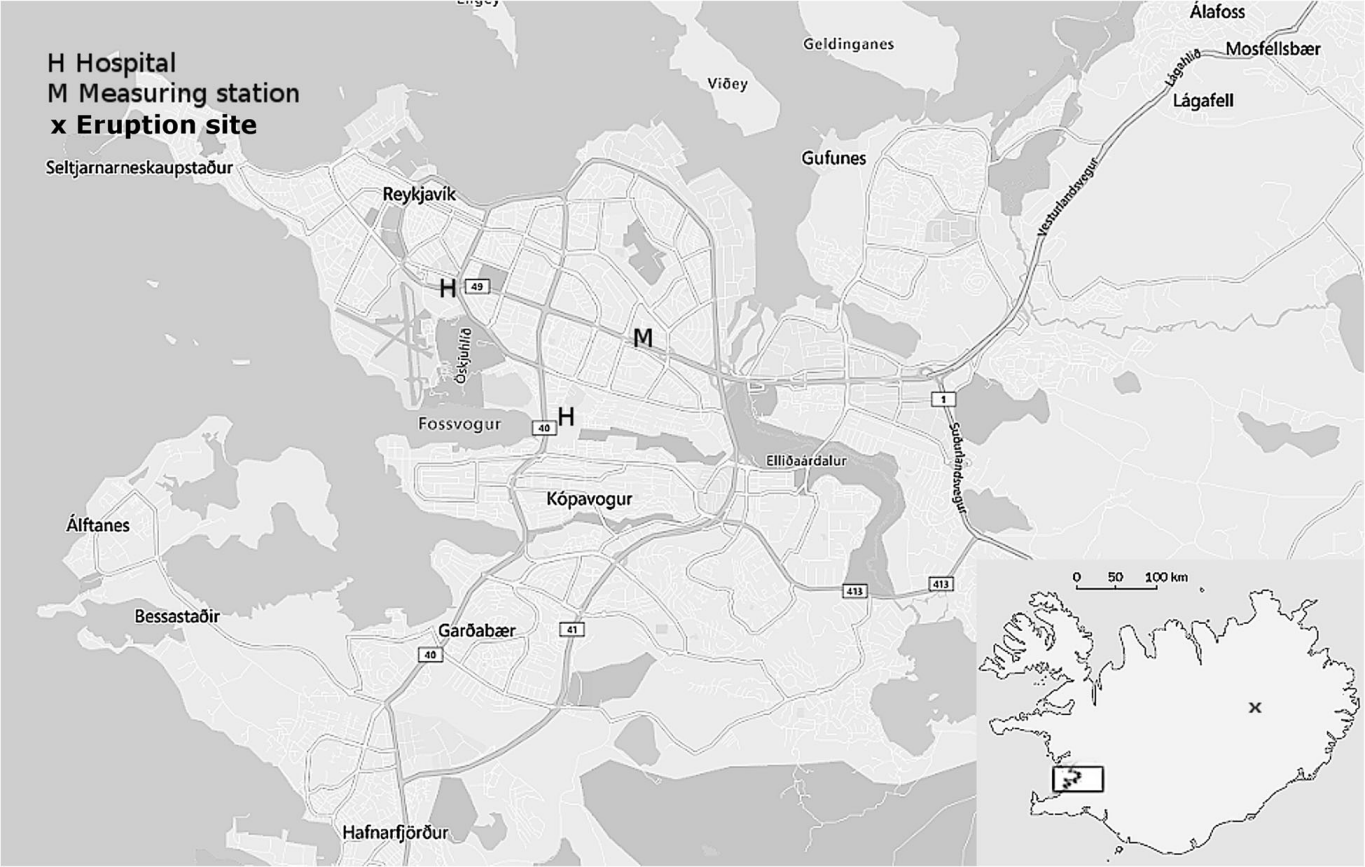
The Icelandic capital region, national location (black box) and eruption site indicated on the inset map (creative commons/google)

In the main analyses, we analysed the number of MD visits in primary care (PCMD) and all HED visits regardless of admission status. As the same bout of illness is likely to result in recurring contacts with the health care system, we included only the first record of an individual’s health care contacts within a 14-day period for the same diagnosis category to avoid exposure misclassification with respect to the timing of the outcome. For each outcome, we constructed daily time series starting 1 January 2010 to 31 December 2014 for the following age groups; children (under 18 years of age), adults (18–64 years), and elderly (age 65 years and above), see data selection in Flow diagrams 1–3 in the supplemental material. We obtained SO_2_, PM_10_ (particle matter with an aerodynamic diameter < 10 μm), and NO_2_ (nitrogen dioxide) data along with meteorological data from the Icelandic Environment Agency’s stationary air pollution monitor located in Reykjavík (Fig. 1) for the study period and constructed a time series of 24-mean values from midnight to midnight.

### Statistical methods

Descriptive statistics were calculated for all exposure and outcome variables for the period before and after the beginning of the eruption (for health data, we used age at date of first occurrence in the data) (see Table 1, see also supplementary material, Flow diagrams 1–3). Correlations of the exposure variables can be found in the supplement (Table S1). We use the t-tests to assess as whether there was a statistically significant difference in concentrations of relevant pollutants and the number of daily health outcome events before, and during the eruption (Table 1 and Table 2).

**Table 1:** Air pollution in Iceland’s capital area before and during the Holuhraun eruption

	Before eruption 2010-01-01 - 2014-08-30 1703 days			During eruption 2014-08-31 - 2014-12-31 123 days			
Pollutant	Mean (SD)	Range	Days with missing data	Mean (SD)	Range	Days with missing data	p*
SO_2_	1.4 (1.1)	0.0–20.6	20	35.7 (71.2)	1.3–418.0	0	< 0.001
PM_10_	20.3 (18.2)	3.8–315.4	19	17.8 (9.1)	5.7–71.5	13	0.010
NO_2_	15.8 (11.3)	0.0–75.9	54	12.0 (8.2)	0.4–41.0	0	< 0.001

*from a *t*-test of means. *SD* Standard deviation

**Table 2:** Demographic characteristics of the study population and daily number of individuals who were dispensed anti-asthma medication (AMD) or utilized health services for respiratory disease before and during the Holuhraun eruption

	Before eruption				During eruption				
	n	%	Events per day, mean (%)	Range	n	%	Events per day, mean (%)	Range	P*
**Anti-asthma medication dispensings (AMD)**									
All	45,473	100%	129.4 (100%)	5–715	11,094	100%	158.4 (100%)	22–301	< 0.001
Female sex n, %)	25,608	56.3%	75.1 (41.8)	2–430	6552	59.1%	93.2 (45.7%)	13–185	< 0.001
Under 18 years of age	14,144	31.1%	28.3 (21.9%)	0–139	2415	21.8%	33.2 (20.9%)	8–68	< 0.001
18–64 years of age	24,117	53.0%	60.7 (46.9%)	3–386	5623	59.7%	75.2 (47.4%)	5–149	< 0.001
65 years and older	7212	15.9%	40.4 (31.2%)	0–202	3056	27.5%	50.1 (31.6%)	0–105	< 0.001
**Primary care center MD visits (PCMD)**									
All	106,528	100%	142.2 (100%)	17–325	15,806	100%	144.8 (100%)	41–259	0.6531
Female sex n, %)	59,429	55.7%	84.6 (59.5%)	9–180	9547	60.4%	87.9 (60.7%)	22–165	0.343
Under 18 years of age	31,240	29.3%	39.2 (27.6%)	3–136	4140	26.2%	38.0 (26.3%)	13–75	0.4019
18–64 years of age	64,840	60.9%	85.9 (60.4%)	12–191	9839	62.2%	89.7 (61.9%)	24–161	0.2951
65 years and older	10,448	9.8%	17.1 (12.0%)	0–48	1827	11.6%	17.1 (11.8%)	0–42	0.9877
**Hospital emergency department visits (HED)**									
All	19,541	100%	18.3 (100%)	1–45	1932	100%	17.5 (100%)	4–34	0.176
Female sex n, %)	9733	49.8%	9.2 (50.5%)	0–29	1000	51.8%	9.0 (51.6%)	2–18	0.527
Under 18 years of age	6811	34.9%	5.7 (30.9%)	0–24	638	33.0%	9.0 (51.6)	0–16	0.640
18–64 years of age	8258	42.3%	7.0 (38.2%)	0–24	758	39.2%	5.5 (31.6%)	1–14	0.269
65 years and older	4472	22.9%	5.7 (31.0%)	0–19	536	27.7%	6.7 (38.0%)	0–13	0.198

*from a *t*-test of means

In the regression analysis of the daily number outcomes during the whole study period, SO_2_ exposure was given as either a) a continuous variable, or b) an indicator value of the 24-h SO_2_ concentration exceeding the air quality guideline value (125 μg/m^3^) [2]. We fitted distributed lag non-linear models (DNLM) to the data [30]. to identify the delay in days (lag days) from exposure to the observed health outcomes (Supplemental Fig. S2) and concentration response (Supplemental Fig. S3 and Fig. S4). We estimated the effects of SO_2_ exposure on the outcome by fitting generalized additive models (GAM) [31].

$$ {\mathrm{Y}}_t\sim \mathrm{Quasipoisson}\ \left({\mu}_t\right)\log\ {\upmu}_t=\upalpha +{\upbeta}_1{\mathrm{SO}}_{2t}+{\upbeta}_2{\mathrm{PM}}_{10}+{\upbeta}_3{\mathrm{NO}}_2+{\upbeta}_5\mathrm{RelativeHumidity}+{\upbeta}_6{I}_{dow}+{\upbeta}_7\mathrm{Strike}+{\upbeta}_8{\mathrm{Y}}_{t- 1}+{\mathrm{s}}_1\left(\mathrm{Temperature}\right)+{\mathrm{s}}_2\left(\mathrm{Day}\ \mathrm{in}\ \mathrm{the}\ \mathrm{time}\ \mathrm{series}\right)+{\mathrm{s}}_3\left(\mathrm{Day}\ \mathrm{of}\ \mathrm{the}\ \mathrm{year},\mathrm{bs}=``\mathrm{cc}"\right) $$

Where Y_*t*_ denotes the daily number of health events, β_1_ denotes the log relative rate of events associated with a 10 μg/m^3^ increase in SO_2_ at lag 0–2. Furthermore, the results are adjusted for co-pollutants and weather (PM_10_, NO_2_, and relative humidity) at the same lag intervals as the main exposure. *I*_*dow*_ is an indicator for day of week and odd holidays, and Strike is an indicator of strike days (used only in analysis of the hospital and primary care data, to indicate days where medical doctors went on strike as part of a labor conflict, which coincided with some high SO_2_ days (See Table S1 for a total list of strike days, and Fig. 1). Several methods of addressing this issue were tested: no adjustment, excluding strike days, or adjusting for the indicator. The latter was found to yield models with a better fit and was used where appropriate. We used an autoregressive term (adjusting for the outcome at lag 1) to improve the autocorrelation of the model residuals [32]. The terms s_1_(Temperature) and s_*2*_(day in the time series) are smoothing functions which allow for non-linearity). The term s_*3*_(day of the year, bs = “cc”) is a b-spline with a penalized cyclical cubic (a spline whose ends match up, and is thus suitable for modeling annual fluctuations) term day of year designed to control for seasonal trend [33]. Results from models with no adjustment for other pollutants (Partially adjusted models) are also presented. Quasipoisson distribution was assumed for all outcomes. All analysis was performed in R Studio [34].

#### Subgroup analysis

We present results from stratified analyses of categories of anti-asthma drugs; 1) adrenergic inhalants (R03A), a large proportion of which are short-acting beta agonists, and 2) other inhalant drugs (R03B), mainly glucocorticoids and anticholinergenics. For PCMD and HED visits, we present results stratified for 1) infectious diseases including acute upper respiratory infections (World Health Organisation International Classification of Disease (ICD) codes J00-J06), influenza and pneumonia (J09-J18), and other acute lower respiratory infections (J20-J22), and 2) obstructive respiratory disease, including chronic obstructive pulmonary disease, COPD, and asthma (J44 and 45). Age-stratified results are also presented.

### Sensitivity analysis

To explore the robustness of the analysis, we analysed the association between all respiratory PCC contacts including phone calls, consultations, and “other” because PCMD visits are subject to availability. To estimate the total impact on the health care systems from increased population morbidity we also analysed all PCC contacts including recurring PCC contacts within 14 days. For HED visits, we performed a sensitivity analysis including only individuals who were admitted for in-patient care (Table S3). Additionally, we performed sensitivity analysis on different lags of SO_2_ for HED admissions, as our exploratory analysis revealed lag-specific effects for different age-categories (Fig. S2c-d, Table S3). In sensitivity analysis of the exposure (Table S4), we wished to exclude the possibility of our results being entirely due to official advice encouraging individuals with respiratory diseases to have sufficient medicine at hand. This was broadcast to the public on days with forecasts of high SO_2_ and it was speculated that some SO_2_-associated increases in AMD could be due to compliance with this advice. As AMD is most often dispensed in larger quantities, a compliance effect would present itself as decreased effect estimates of SO_2_ after the first days or week of warnings as regular users have filled their supplies. Thus, present results from analyses where we 1) excluded the first day, then 2) the first week, from the time series (Table S4). To eliminate any confounding effect of air pollution from other volcanic eruptions ((Eyjafjallajökull 2010 and Grímsvötn 2011) that impacted air quality during the study period (Daily mean of key air pollutants and events are indicated in Fig. S1) [35] we reanalysed AMD, PCMD and HED excluding the years 2010 and 2011 (Table S4). Also, we performed analyses allowing for non-linearity of both lag structure, and concentration of SO_2_ and present those results in the supplement (Fig. S2 and Fig. S3). Furthermore, we investigated lag-responses of sub-categories of respiratory disease (Fig. S4) and effects of longer lags (Fig. S5).

## Results

**Fig. 2:**
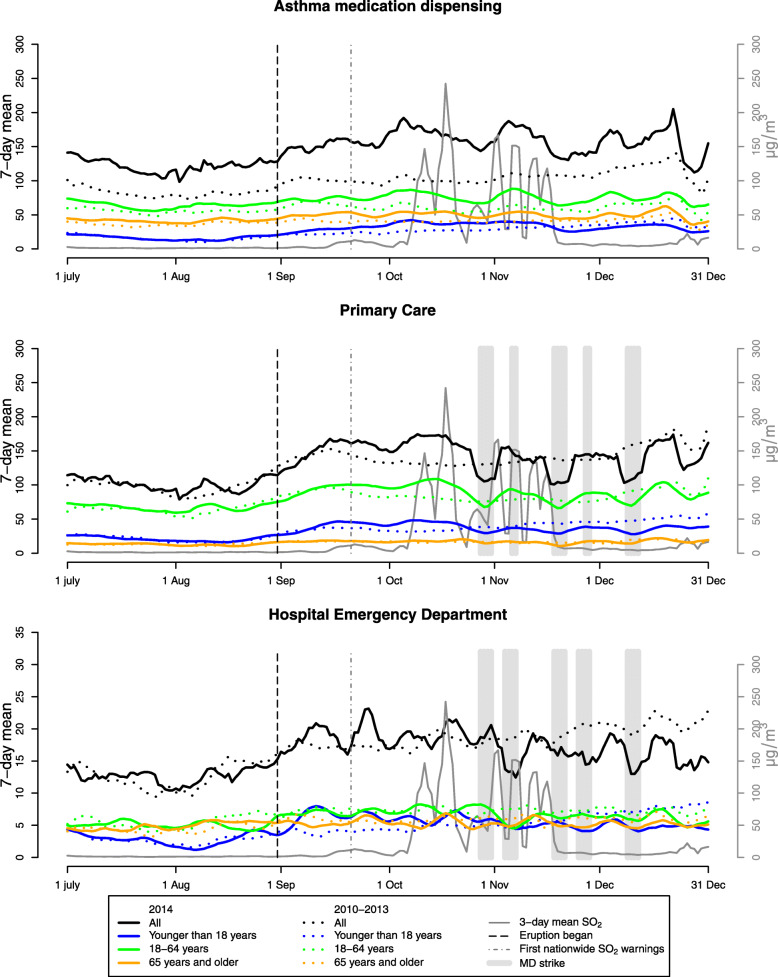
Respiratory health outcomes during the exposed period July to December 2014 (solid lines) and the unexposed previous years 2010–2013 (broken lines) for Capital area anti-asthma medication dispensing (top), primary care MD visits (middle) and hospital emergency department visits (bottom) for respiratory diseases as 7-day running means (right side axis). SO_2_ concentration (3-day running means) in grey (unit on left side axis), black vertical, broken line mark the eruption date, grey vertical broken line marks day of first nation-wide SO_2_ warning. Grey shaded areas indicate MD strike in PCC and HED

The daily mean SO_2_ concentrations in Iceland’s capital area were low or moderate until the Holuhraun eruption began 30 August 2014. After the eruption began, 24-h SO_2_ concentrations surpassed the air quality guideline limit of 125 μg/m^3^ on ten days. Both mean and median concentrations were significantly higher than before the eruption; mean 35.7 μg/m^3^ (SD 71.2) vs mean 1.4 μg/m^3^ (SD 1.1) (Table 1, see also Fig. 2 and Fig. S1), and the correlations between air pollutants were altered substantially after the eruption (Table S1).

Before the eruption, an average of 129.4 individuals per day were registered purchasing (=were dispensed) AMD. There were 56.3% females and the mean age was 35.7 (SD 22.9) years. In primary care, the most common form of contact was GP visits (61%) followed by phone calls (33%), the daily mean number of individuals with an MD visits in primary care (PCMD) for respiratory disease was 142.2, the mean age was 32.8 (SD 22.5) years and 60.9% were women. In HED, the daily mean number of individual visits for respiratory health outcomes were 18.3, the mean age was 36.3 years (SD 29.4) and 49.8% were women. After the eruption began, 158.4 individuals per day were dispensed AMD, the mean age was 44.5 (SD 26.3) years. 144.8 individuals per day visited an MD in primary care for respiratory disease, the mean age was 35.0 years (SD 26.6), 60.4% were women. In HED, 17.5 individuals attended for respiratory disease, the individuals had a mean age of 39.4 years (SD 30.5) and 51.8% were women (Table 2). Comparing the daily number of events, only mean daily number of individuals with AMD was significantly increased compared with the reference period (129.4 vs 158.4, *p* < 0.001). Restricting this analysis to only the time of the year where the eruption was ongoing (using September to December in the years 2012 and 2013 where there were no volcanic eruptions) we found that AMD was significantly increased 138.2 vs 158.4, *p* = 0.0035). Neither total nor age categories of HED and PCMD MD visits were significantly higher or lower during the eruption period as a whole compared to the period before (Table 2, Fig. 2).

We found that for AMD, the overall time trend adjustment without fixed degrees of freedom showed signs -of overfitting and so, comparing the model fits (R^2^ and explained deviance), 4 degrees of freedom was selected as the optimal value for the time trend spline in analyses of AMD. For PCMD and HED, the degrees of freedom were not fixed. After inspecting the residuals for signs of drift, we were confident that the time trends adjust for the population increase during the study period.

In both partially (model 1) and fully adjusted (model 2) models, SO_2_ was associated with increased number of AMD (model 2) by 0.99% (95% CI 0.39–1.58%) per 10 μg/m^3^ SO_2_. High SO_2_–days, with concentrations exceeding the air quality guideline of 125 μg/m^3^ were associated with a statistically significant increase in AMD by 10.9% (95% CI 2.1–19.6%) at lag 0–2 (Table 3). For PCMD and HED, the strike indicator for days where MDs were on strike was associated with fewer outcome events (as fewer MDs were working). The inclusion of the strike indicator variable improved the model fit and was an effect modifier of SO_2_, increasing the effect estimates, and thus it was added to the following PCMD and HED models. In primary care, SO_2_ was associated by an increase in PCMD visits by 1.26% (95% CI 0.72–1.80%) per 10 μg/m^3^ SO_2_ at lag 0–2. For PCMD, exposure to SO_2_ over 125 μg/m^3^ was associated with increases by 17.2% (95% CI 10.0–24.4%) at lag 0–2. For HED visits, only adjusted results (model 2) of continuous SO_2_ exposure at lag 0–2 yielded significant associations associated with increase (Fig. S2d) in total HED by 1.02% (95% CI 0.02–2.03%) per 10 μg/m^3^. There were no statistically significant associations between high SO_2_ days and total HED (Table 3). The effect estimates from the partially adjusted models (model 1) were within the confidence intervals of the model 2 fully adjusted results (~ 10 percentage point change), except for HED visits which increased from 0.83% (95% CI − 0.12 – 1.78%) in the unadjusted model 1 to 1.02% (95% CI 0.02–2.03%) per 10 μg/m^3^ SO_2_ in model 2 (Table 3). Fully adjusted model results are presented from here.

**Table 3:** Percent excess risk associated with daily SO_2_ exposure at lag 0–2 (for exposure as a continuous variable, left, and as an indicator for days with pollution levels above the air quality guideline value, right) and respiratory health outcomes in the capital area of Iceland

	SO_**2**_ (per 10 μg/m^**3**^)			SO_**2**_ levels > 125 μg/m^**3**^		
	%	95% CI		%	95% CI	
**Asthma medication (AMD)**						
Model 1	**0.89%**	**0.29%**	**1.49%**	**10.5%**	**1.8%**	**20.0%**
Model 2	**0.99%**	**0.39%**	**1.58%**	**10.9%**	**2.1%**	**19.6%**
**Primary care MD visits (PCMD)**						
Model 1	**1.18%**	**0.68%**	**1.69%**	**16.9%**	**9.2%**	**25.0%**
Model 2	**1.26%**	**0.72%**	**1.80%**	**17.2%**	**10.0%**	**24.4%**
**Hospital Emergency Department (HED)**						
Model 1	0.83%	-0.12%	1.78%	11.7%	−1.4%	26.7%
Model 2	**1.02%**	**0.02%**	**2.03%**	9.4%	−3.8%	22.6%

Model 1: Adjusted for season, time trend, day of week, odd holidays, the outcome at lag 1, temperature, and relative humidity. *n* = 1606

Model 2: Adjusted for season, time trend, day of week, odd holidays, the outcome at lag 1, temperature, relative humidity, NO_2_, PM_10_ at the same lags as SO_2_. *n* = 1529

In subgroup analysis (Table 4), the dispensing of short acting bronchodilator medication was associated with SO_2_ exposure by 1.09% (95% CI 0.49–1.70%) per 10 μg/m^3^ SO_2_, and 13.7% (95% CI 4.5–23.7%) after high SO_2_ days. SO_2_ exposure was not significantly associated with dispensing of long-acting corticosteroid medication. SO_2_ exposure was associated with increased PCMD visits for respiratory infection diagnoses by 1.12% (95% CI 0.54–1.71%) and obstructive pulmonary disease by 2.08% (95% CI 1.13–3.04%) per 10 μg/m^3^ at lag 0–2. and PCMD visits for %). Following high SO_2_ days, the estimated increase in PCMD visits were 15.8% (95% CI 7.2–25.1%) and 28.4% (95% CI 12.8–46.2%) for respiratory infections and obstructive disease, respectively. For HED visits, SO_2_ exposure was not associated with significant increases in visits due to respiratory infections or asthma or COPD although all estimates were numerically indicative of a positive effect (Table 4, Fig. S4).

**Table 4:** Percent excess risk associated with SO_2_ exposure at lag 0–2 (both for exposure as a continuous variable and for days with pollution levels above the air quality guideline value) and changes in respiratory health in diagnosis subcategories in primary care and hospital emergency departments in the capital area of Iceland

	Mean (SD)	SO_**2**_ (per 10 μg/m^**3**^)^a^			SO_**2**_ levels > 125 SO_**2**_ μg/m^**3**^		
		%	95% CI		%	95% CI	
**Anti-asthma medication**							
Short-acting β-agonist (R03A)	88 (47.2)	**1.09%**	**0.49%**	**1.70%**	**13.7%**	**4.5%**	**23.7%**
Long-acting (R03B)	38.7 (21.4)	0.74%	−0.03%	1.52%	8.7%	−2.2%	20.7%
**Primary care MD visits (PCMD)**							
Respiratory infections (J0-J2)	108.6 (46.2)	**1.12**	**0.54%**	**1.71%**	**15.8%**	**7.2%**	**25.1%**
Asthma and COPD (J44–45)	15.6 (9.6)	**2.08%**	**1.13%**	**3.04%**	**28.4%**	**12.8%**	**46.2%**
**Hospital emergency department visits (HED)**							
Respiratory infections (J0-J2)	9.3 (4.3)	1.09%	−0.22%	2.41%	7.9%	−60.9%	27.8%
Asthma and COPD (J44–45)	4.3 (2.7)	0.95%	−0.99%	2.93%	3.3%	−19.8%	33.2%

^a^Adjusted for season (spline), time trend (spline), day of week, odd holidays 1, temperature (spline), relative humidity, NO_2_, PM_10_ at the same lags as SO_2_, and the outcome at lag

In analysis stratified by age, the estimated association between SO_2_ and AMD exposure at lag 0–2 were highest in children, and lower in elderly, however, all confidence intervals overlapped in both partially and fully adjusted models (Table 5). In PCMD, the effect estimates were highest in adults, and was not statistically significant in elderly.

**Table 5:** Percent excess risk in age-specific respiratory health outcomes associated with SO_2_ exposure at lag 0–2 (both for exposure as a continuous variable and for days with pollution levels above the air quality guideline value)

	SO_**2**_ (per 10 μg/m^**3**^)			SO_**2**_ levels > 125 μg/m^**3**^		
	%	95% CI		%	95% CI	
**Anti-asthma medication (AMD)**						
Children 0–17	**1.48%**	**0.61%**	**2.35%**	**20.2%**	**7.8%**	**32.6%**
Adults 18–65	**0.86%**	**0.20%**	**1.53%**	**11.3%**	**1.8%**	**20.9%**
Elderly > 65	**0.90%**	**0.14%**	**1.67%**	9.8%	−1.4%	20.9%
**Primary care MD visits (PCMD)**						
Children 0–17	**1.24%**	**0.40%**	**2.08%**	**14.2%**	**3.0%**	**25.5%**
Adults 18–65	**1.21%**	**0.63%**	**1.79%**	**18.5%**	**10.6%**	**26.3%**
Elderly > 65	**1.11%**	**0.14%**	**2.09%**	11.8%	−2.0%	25.6%
**Hospital Emergency Department (HED)**						
Children 0–17	1.22%	−0.62%	3.10%	7.0%	−18.7%	32.7%
Adults 18–65	0.52%	− 0.99%	2.06%	0.0%	−20.7%	20.7%
Elderly > 65	1.01%	−0.62%	2.66%	19.7%	−1.8%	41.2%

All results are adjusted for season, time, day of week, odd holidays, the outcome at lag1, and MD strike for PCC and HED, relative humidity, temperature, PM_10_, and NO_2_. *n* = 1788

Changing the outcome to include all primary care contacts (also phone calls) and further including the recurring contacts for the same diagnostic category within 14 days only marginally alter the results (17.2% vs 17.4% vs 20.3%) for high SO_2_ levels (Table S3).

Considering effects on HED of SO_2_ exposure at lag 2–4, the effect estimated in children increased numerically from 1.22% (95% CI -0.62 – 3.10%) to 2.21% (95% CI 0.30–3.97%) however, the confidence intervals overlapped and statistical power was low (Table S3).

Confining the analysis of HED to those wwo were admitted, there were no associations between SO_2_ exposure at lag0–2, but admission in children were increased at lag 2–4 by 6.6% per 10 μg/m^3^ SO_2_ (Table S3).

Excluding the first day in a series of high SO_2_ days from the exposure variable yielded very similar estimated effects of SO_2_ exposure to the main analysis for AMD, but slightly lower for PCMD (Table S4). Excluding the whole week after the first high SO_2_ concentration day, effect estimates for PCMD visits were higher in elderly compared with the main analysis (Table S4). Excluding the years 2010 and 2011 yielded statistically significant effect estimates for total AMD and PCMD, but only HED in elderly was significantly increased (Table S4). In the analysis of HED admissions rather than all HED visits, all effect estimates were positive, but there were no significant associations between SO_2_ and HED at lag 0–2. When increasing the lag-period to 20 days we observed a second increase in PCMD at lag 16 after the initial SO_2_ exposure, and at lag 11–13 for HED in young people and adults (Fig. S4).

## Discussion

Although SO_2_ pollution levels were overall higher after the Holuhraun eruption began (Table 1), a crude comparison of respiratory health care utilisation in Iceland’s capital area before and after the beginning of the Holuhraun eruption (Table 2) showed that only AMD increased significantly compared to the corresponding months in previous years, as well as compared to the total control period.. However, in time series regression analysis (Table 3), SO_2_ concentrations were associated with increased AMD, PCMD and HED for respiratory causes at lag 0–2. Analysing the association with SO_2_ levels which exceeded the 24-h health limit (125 μg/m^3^) to quantify the risk associated with high levels yielded similar results, and spline plots suggest a linear dose-response curve (Table 3, see also Fig. S3). Stratifying AMD into subgroups, only dispensing of short-acting medications were significantly increased, indicating that dispensing of short-acting drugs are a more sensitive indicator of an immediate need for symptom relief, although the use of short-acting drugs are not specific to asthma disease (Table 4). In PCMD we found that PCMD for respiratory infections and obstructive lung disease were increased and the increase in obstructive lung disease suggested that individuals with this diagnosis were increasingly in need of care following SO_2_ exposure (Table 4). For COPD diagnoses in HED, our results indicated a positive association, but it was lower than for PCMD, and the results did not reach statistical significance.

Stratifying by age-categories, the results indicated that for AMD, we observed with highest risk estimates with SO_2_ exposure in individuals under 18, for PCMD, adults had highest risk, and for HED the highest risk was in individuals 65 and older (Table 5), except for lag2–4, where it was higher in individuals under 18 (Table S3). However, the confidence intervals overlapped, and the association between SO_2_ and HED in elderly was not significant after adjusting for other pollutants than SO_2_ (Table 5).

Exploring definitions of outcomes in primary care in sensitivity analyses, the tendency towards increasing effect estimates in elderly in analyses of all contacts and recurring contacts (Table S3) rather than non-recurring MD visits suggests that the care burden is higher in this group, but the confidence intervals overlapped with those in the main analysis (Table 5). In HED admissions, a high effect estimate was seen for both continuous SO_2_ and high SO_2_ days in children at lag2–4, but the confidence intervals were wide (Table S3).

In the regression analysis, the choice of lags were based on inspection of lag structures generated by lag-splines (Fig. S2). For AMD and PCMD visits, the observed effects of SO_2_ exposure occurred during the same day and up to 2 days after a peak in SO_2_ concentration (Figs. S2a-b). However, for HED visits in elderly, the observed effects occurred already at lag 0 (Fig. S2c-d). A possible explanation for this is that primary care is the first point of contact for most individuals and hospital care is sought only after other care options have been exhausted, but for elderly, hospital care is sought immediately, possibly due to a more severe presentation of symptoms or complications due to underlying diseases, as illustrated by a significant association between SO_2_ and HED in elderly before adjusting for other pollutants at lag0–2 (Table S3), and significant association only with HED in children at lag 2–4 (Table S3). Unfortunately, we did not have access to information about comorbidities and were unable to adjust for this.

In the sensitivity analysis to investigate the influence of compliance with official advice, the effect of initial warnings of volcanic gas exposure appeared to be limited, as excluding the first day or week of high SO_2_ yielded similar estimates for AMD and PCC visits as the main analysis, although there was some loss of statistical power (Table S4). We speculate that since the increases in AMD and PCC persisted after the first exposed day and weeks, this indicates that the observed increases in health care utilisation reflect actual increased respiratory morbidities rather than merely adherence to official advice (Supplemental Table S4). Excluding the years 2010 and 2011 from the analysis to exclude effects of volcanic ash from Eyjafjallajökull and Grímsvötn yielded results within confidence intervals of our main analysis (Table S4). Investigating longer lags, we observed statistically significant increases in total PCMD and HED visits in young and adults at more than 15 days delay, but in both cases, these follow periods of decreases in outcomes, and are challenging to interpret (Fig. S5).

Although it has been suspected that the complex mixture of a volcanic plume [7] could have different health effects than merely SO_2_, we found that our results were consistent with concentration-response functions found in studies from urban settings; e.g. respiratory mortality rates were estimated to increase by 2.4% per 27 μg/m^3^ SO_2_ (Li et al. 2017), corresponding to a point estimate of 0.88% per 10 μg/m^3^ SO_2_, lower, but within the confidence interval of the estimated increases of HED found in our study, namely 1.02% (95% CI 0.02–2.03%) per 10 μg/m^3^. In previous studies of SO_2_ exposure during volcanic eruptions, the SO_2_ concentration on Miyakejima Island increased after Mount Oyama erupted in 2000 meaning that residents and aid workers returning to the island from 2005 and onwards were exposed. Children living on the island who were exposed to daily mean concentration of 125 μg/m^3^ SO_2_, had increased rates of wheezing [36]. In follow-up studies, permanent residents of Miyakejima Island (*n* = 168) who lived in areas with high SO_2_ exposure reported increased rates of cough and wheeze [13], both symptoms of asthma. None of the studies of people exposed at Miyakejima Island found adverse effects on lung function [12, 14, 36].

Regarding individual susceptibility, we observed the highest effect estimates for PCMD visits for asthma and COPD (Table 4), indicating that individuals with these diseases are at increased risk, which is. Hyper-responsiveness to SO_2_ has previously been reported as common (20–25%) in individuals with positive asthma test (methacholine test), indicating that they may be particularly vulnerable to severe SO_2_ exposure [4]. Non-smokers have previously been reported as being at risk of symptoms after volcanic SO_2_ exposure [11], but unfortunately, smoking status was not available in our data.

Respiratory infections were increased in our study and similarly rates of cough and acute pharyngitis diagnosed at a clinic in SO_2_ –exposed communities near The Kilauea Volcano in Hawaii increased after a eruption activity increased in 2008 [18]. Respiratory infection diagnoses in PCMD were statistically significantly associated with SO_2_, a similar association has previously been reported for hospital visits for upper respiratory tract infections [37]. Volcanic eruptions have been associated with altered rates of other health outcomes such as accidents and mortality [38], but these fell outside the scope of this study.

Our study employs a time series design were individual level risk factors are not time-dependent and thus should not confound the association between short term exposure to air SO_2_ and health outcomes, leaving bias due to unmeasured confounders, seasonal variation, and other intermittent exposures as main concerns. Previous studies of health effects of air pollution in Iceland have yielded lower effect estimates of daily air pollution on morbidity than the current study [39, 40], other air pollution exposure types are thus not likely to bias the results. This includes H_2_S, which has a low correlation (< 0.1) with the exposure of interest during the study period (data not shown). During the reference period, there were two ash-rich volcanic eruptions, in Eyjafjallajökull 2010 and Grímsvötn 2011. While ash from Eyjafjallajökull had local respiratory health effects [24] and there may have been adverse health effects in the capital area [40], neither eruption had significant SO_2_ emissions and sensitivity analysis excluding this period yielded results within the confidence interval of the main study results (Supplemental Table S5). The exposed period October and November of 2014 did not coincide with any viral respiratory illnesses (e.g. influenza and RS-virus) epidemic [41, 42], but it did coincide with the MD labour conflict which resulted in lower PCMD and HED attendance during those days. Hence, results from the analysis comparing the eruption period with the time before may have underestimated of true effect of the SO_2_ from the eruption.

SO_2_, PM_10_ and NO_2_ data was missing for a number of days which were excluded from the analysis. As the volcanic plume effectively changed the chemical composition of the atmosphere during the eruption period (Supplemental Tables S1a-b), the correlations of SO_2_ with other air pollutants were altered after the eruption. Results that were adjusted for other pollutants were nearly identical to the main analysis (Table 3), indicating that the effect of the very high concentrations of SO_2_ was unambiguous.

It is a strength of the current study that health data were collected prospectively from population-wide registers, which minimizes the risk of information bias from individuals knowing their exposure status. Although the exposure would have been known to the public for at least part of the exposed period but we attempt to address this source of bias in the unadjusted analysis and found only moderate changes to the results (Table S3). As a study outcome, we use dispensing of asthma-medication, a novel and more sensitive proxy for respiratory health in a population [28] than primary care attendance and hospital visits. It targets individuals who are already sensitive to poor air quality [26, 29] who in most cases have contact with the health care system. A limitation with register-data is that the data are not collected for research purposes and diagnoses given in the health care system could be biased and lead to overestimation as medical professionals assume respiratory outcomes to be more likely during the eruption. However, as the estimated effect are similar across all respiratory outcomes we conclude that this source of bias is not likely to explain our findings. By defining exposure status based on residential postcode we make several assumptions, firstly, that the whole area is equally exposed, and secondly, that the study participants are physically near their homes. However, these sources of bias would result in wider confidence intervals or bias the results toward the null. Reykjavík and the Icelandic capital area was exposed to SO_2_ concentrations above 125 μg/m^3^ during a total of ten days which occurred mostly during October and November 2014. This limits the statistical power of the study and our options for further analysis, as does the fact that our study period did not extend until after the eruption, meaning that we cannot fully assess the complete impact of the health impact from the eruption on the population from our results.

## Conclusions

In conclusion, this comprehensive study with prospectively collected data on volcanic SO_2_ air pollution exposure and respiratory health outcomes, is the first to firmly establish an association between spikes of high SO_2_ concentrations and respiratory outcomes in the general population, and specifically in individuals with prevalent respiratory disease. These findings emphasize the need for attention from authorities and susceptible individuals during times of volcanic eruptions.

## Supplementary Information

**Additional file 1.**

## Abbreviations

AMD: Asthma medication dispensing
COPD: Chronic obstructive pulmonary disease
HED: Hospital emergency department
PCC: Primary care contacts
PCMD: Primary care MD visits
SO_2_: Sulfur dioxide
PM_10_: Particle matter less than 10 μg/m^3^
