# Supplementary material for: Severe volcanic SO2 exposure and respiratory morbidity in the Icelandic population – a register study
Source: Environ Health. 2021 Feb 27;20:23. doi: 10.1186/s12940-021-00698-y (PMC7916308; doi:10.1186/s12940-021-00698-y)
Supplement: Supplementary file 1 — Additional file 1. [file 12940_2021_698_MOESM1_ESM.docx]

Table of Contents

Table of Contents 1

Supplementary tables 3

Table S1a-b Exposure correlation matrix 3

a) Before eruption (1 Jan 2010-august 31 2014) 3

b) After eruption (31 August 2014-31 December 2014) 3

Table S2 Dates with high SO2 and strikes in primary care and hospital 4

Table S3 Sensitivity analyses: Outcome variable definitions and lag-days 5

Table S4 Sensitivity analyses: Exposure definitions and subset analyses 6

Supplementary figures 8

Figure S1 Time series of daily exposure and outcomes 2010-2014. 8

Figure S2 a-d High SO_2_ (>125 ug/m^3^) days and associations with respiratory health outcomes across lags 0-10 (plotted using DLNM) 10

Figure S3 a-c Concentration-response for SO_2_ and health outcomes (plotted using DLNM) 11

Figure S4 a-d Associations between high SO_2_ days (>125 ug/m^3^) and subcategories of PCMD and HED (plotted using DLNM) 12


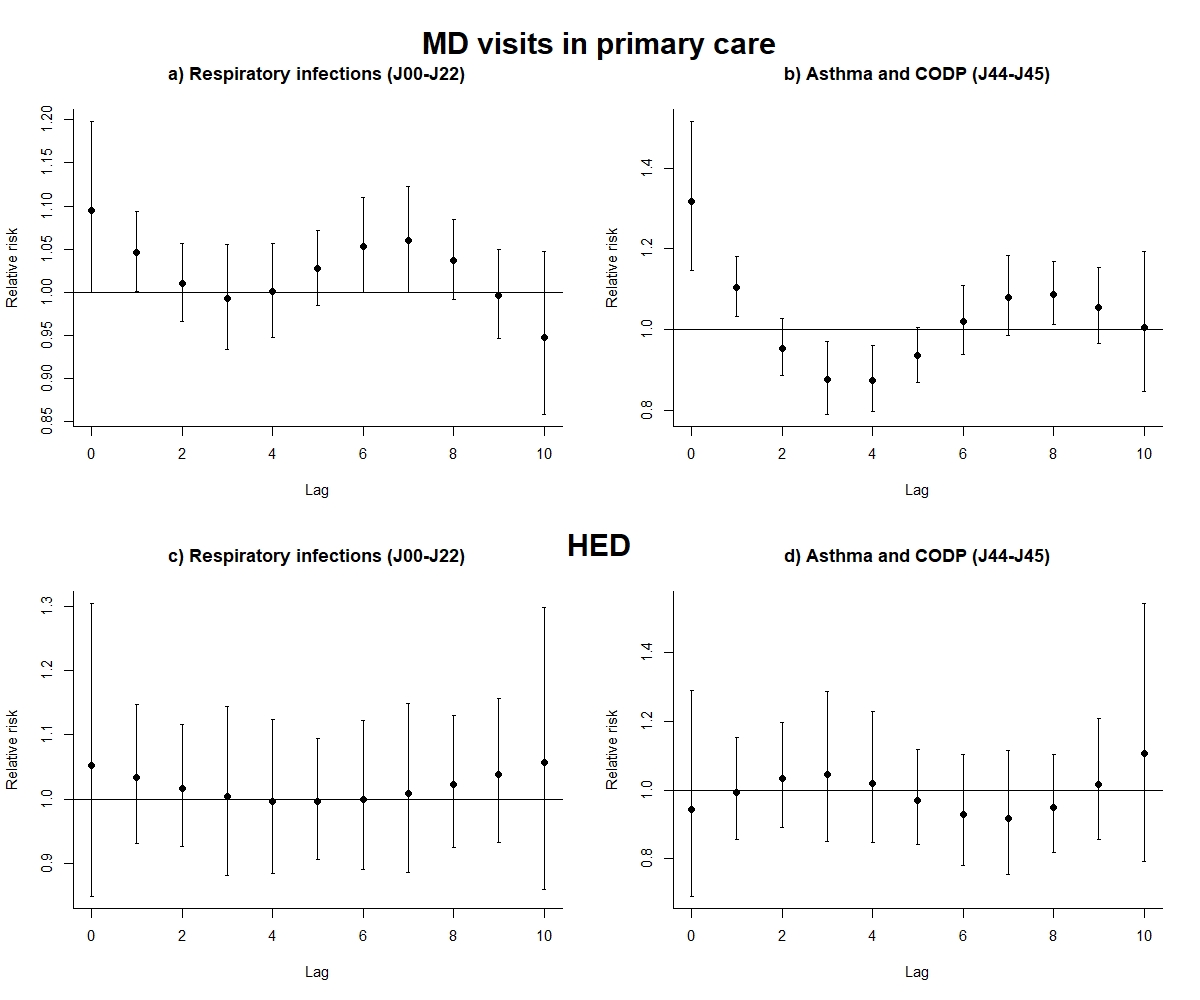
 Figure S5 a-d Lag-associations to lag 20 (plotted using DLNM) 12

Data selection flow diagrams 14

Flow diagram 1: Anti-asthma medication dispensing (AMD data) 14

Flow diagram 2: Primary care center MD visits (PCMD data) for respiratory diagnoses 15

Flow diagram 3: Hospital emergency department visits (HED data) for respiratory diagnoses 16

# Supplementary tables

## Table S1a-b Exposure correlation matrix

### Before eruption (1 Jan 2010-august 31 2014)

| n=1466 | SO_2_ | PM_10_ | NO_2_ | Temperature | Relative humidity |
| --- | --- | --- | --- | --- | --- |
| SO_2_ | 1 |  |  |  |  |
| PM_10_ | -0.006 | 1 |  |  |  |
| NO_2_ | 0.429* | 0.002 | 1 |  |  |
| Temperature | -0.092* | -0.155* | -0.502* | 1 |  |
| Relative humidity | 0.103* | -0.164* | 0.103* | 0.065* | 1 |

### After eruption (31 August 2014-31 December 2014)

| n=109 | SO_2_ | PM_10_ | NO_2_ | Temperature | Relative humidity |
| --- | --- | --- | --- | --- | --- |
| SO_2_ | 1 |  |  |  |  |
| PM_10_ | 0.403* | 1 |  |  |  |
| NO_2_ | 0.075 | 0.400* | 1 |  |  |
| Temperature | -0.036 | -0.129 | -0.620* | 1 |  |
| Relative humidity | -0.556* | -0.352* | -0.050 | 0.081 | 1 |

* p<0.05

## Table S2 Dates with high SO2 and strikes in primary care and hospital

| **Date** | **Daily mean SO_2_ > 125 µg/m^3^** | **Strike (Primary care)** | **Strike**  **(Hospital)** |
| --- | --- | --- | --- |
| 2014-10-08 | yes | - | - |
| 2014-10-10 | yes | - | - |
| 2014-10-14 | yes | - | - |
| 2014-10-15 | yes | - | - |
| 2014-10-16 | yes | - | - |
| 2014-10-27 | - | yes | yes |
| 2014-10-28 | - | yes | yes |
| 2014-10-29 | yes | yes | yes |
| 2014-10-30 | yes | yes | yes |
| 2014-11-03 | - | yes | yes |
| 2014-11-04 | yes | yes | yes |
| 2014-11-05 | - | yes | yes |
| 2014-11-06 | - | yes | yes |
| 2014-11-09 | yes | - | yes |
| 2014-11-13 | yes | - | yes |
| 2014-11-17 | - | yes | yes |
| 2014-11-18 | - | yes | yes |
| 2014-11-19 | - | yes | yes |
| 2014-11-20 | - | yes | yes |
| 2014-11-26 | - | yes | yes |
| 2014-11-27 | - | yes | yes |
| 2014-12-08 | - | yes | yes |
| 2014-12-09 | - | yes | yes |
| 2014-12-10 | - | yes | yes |
| 2014-12-11 | - | yes | yes |
| 2014-11-24 | - | - | yes |
| 2014-11-25 | - | - | yes |

## Table S3 Sensitivity analyses: Outcome variable definitions and lag-days

Association between SO_2_ as both continuous variables and as indicator of days with high levels (SO_2_>125 µg/m^3^) and changes in anti-asthma medication dispensing, respiratory primary care center contacts including recurring contacts and hospital emergency admissions due to respiratory illness.

|  |  | **SO_2_ (per 10 µg/m^3^)** | | | | **SO_2_ levels >125 ug/m^3^** | | | | |
| --- | --- | --- | --- | --- | --- | --- | --- | --- | --- | --- |
|  | **Mean (sd)** | **%** | | **95% CI** | | | | **%** | 95% CI | |
| **Primary care – All contacts (lag 0-2)** | | | | | | | | | |  |
| All | 192.3 (91.2) | 1.15% | 0.64% | | 1.67% | | 17.4% | | 9.4% | 26.0% |
| Children 0-17 | 46.2 (22.1) | 1.01% | 0.20% | | 1.82% | | 12.4% | | 0.6% | 25.5% |
| Adults 18-65 | 116.4 (56.0) | 1.14% | 0.59% | | 1.68% | | 19.1% | | 10.5% | 28.4% |
| Elderly >65 | 29.7 (19.1) | 1.28% | 0.49% | | 2.06% | | 17.8% | | 5.3% | 31.8% |
| **Primary care - including recurring contacts (lag 0-2)** | | | | | | |  | |  |  |
| All |  | 1.29% | 0.79% | | 1.80% | | 20.3% | | 12.2% | 28.9% |
| Children 0-17 |  | 0.40% | -0.13% | | 0.93% | | 4.2% | | -2.9% | 11.9% |
| Adults 18-65 |  | 1.14% | 0.59% | | 1.68% | | 19.1% | | 10.5% | 28.4% |
| Elderly >65 |  | 1.37% | 0.86% | | 1.87% | | 21.4% | | 13.3% | 30.1% |
| **Hospital emergency department visits (HED, lag 2-4)** | | | | | | | | |  |  |
| All |  | **1.06%** | **0.03%** | | **2.10%** | | 9.7% | | -4.0% | 25.5% |
| Children 0-17 |  | **2.12%** | **0.30%** | | **3.97%** | | 25.3% | | -2.4% | 60.8% |
| Adults 18-65 |  | 0.50% | -1.09% | | 2.10% | | -0.3% | | -19.2% | 23.0% |
| Elderly >65 |  | 0.28% | -1.44% | | 2.03% | | 5.2% | | -16.1% | 31.9% |
| **Admissions from Hospital emergency department (lag 0-2)** | | | | | | | | | |  |
| All |  | 0.76% | -0.81% | | 2.34% | | 15.6% | | -11.3% | 50.6% |
| Children 0-17 |  | 3.76% | -0.75% | | 8.27% | | 65.9% | | -34.1% | 317.6% |
| Adults 18-65 |  | 0.88% | -1.75% | | 3.50% | | 5.4% | | -35.5% | 72.1% |
| Elderly >65 |  | 0.65% | -1.22% | | 2.51% | | 13.9% | | -17.7% | 57.7% |
| **Admissions from Hospital emergency department (lag 2-4)** | | | | | | | | | |  |
| All |  | 1.71% | -0.18% | | 3.60% | | 17.8% | | -9.4% | 53.1% |
| Children 0-17 |  | **6.61%** | **1.27%** | | **11.96%** | | **157.4%** | | **10.3%** | **500.7%** |
| Adults 18-65 |  | 2.09% | -0.80% | | 4.99% | | 22.5% | | -21.8% | 92.1% |
| Elderly >65 |  | 1.08% | -1.20% | | 3.35% | | 6.9% | | -23.0% | 48.3% |

Models are adjusted for NO_2_, PM_10_, and relative humidity at the same lags as SO_2_, odd holidays, day of week, the outcome at lag1, and with splines for temperature, season, and overall time trend. n=1547 for all.

## Table S4 Sensitivity analyses: Exposure definitions and subset analyses

Association between SO_2_ as both continuous variables and as indicator of days with high levels (SO_2_>125 ug/m^3^) and changes in anti-asthma medication dispensing, respiratory PCMD and visits and hospital emergency visits and admissions

|  | **SO_2_ (per 10 µg/m^3^)** | | |  | | | **SO_2_ levels >125 ug/m^3^** | | | |  |
| --- | --- | --- | --- | --- | --- | --- | --- | --- | --- | --- | --- |
|  | **%** | **95% CI** | | | **%** | | | **95% CI** | | | |
| **Excluding the first day in series of consecutive high SO_2_ exposure days**  Asthma medication (AMD, lag 0 – 2) | | | | | | | | | | |  |
| All | **1.37%** | **0.70%** | **2.05%** |  | | **13.5%** | | | **2.9%** | **25.3%** |  |
| Children 0 – 17 | **1.31%** | **0.31%** | **2.32%** |  | | **17.3%** | | | **1.5%** | **35.7%** |  |
| Adults 18 – 65 | 0.66% | -0.10% | 1.42% |  | | 11.4% | | | -0.2% | 24.4% |  |
| Elderly >65 | **1.25%** | **0.43%** | **2.07%** |  | | **16.9%** | | | **3.1%** | **32.6%** |  |
| Primary care MD visits (PCMD, lag 0-2) | | | |  | |  | | |  |  |  |
| All | **1.22%** | **0.06%** | **2.38%** |  | | **18.2%** | | | **8.8%** | **28.3%** |  |
| Children 0-17 | **2.02%** | **0.33%** | **3.74%** |  | | **17.0%** | | | **3.1%** | **32.7%** |  |
| Adults 18-65 | 0.87% | -0.40% | 2.16% |  | | **16.7%** | | | **6.7%** | **27.6%** |  |
| Elderly >65 | 0.51% | -1.68% | 2.74% |  | | **24.4%** | | | **7.0%** | **44.7%** |  |
| **Excluding the first week of high SO_2_ exposure**  Anti – asthma medication (AMD, lag 0 – 2) | | | | | | | | | | |  |
| All | **0.95%** | **0.25%** | **1.65%** |  | | 9.0% | | | -0.5% | 19.5% |  |
| Children 0 – 17 | **1.48%** | **0.47%** | **2.50%** |  | | 11.2% | | | -3.1% | 27.5% |  |
| Adults 18 – 65 | **0.95%** | **0.17%** | **1.75%** |  | | 10.4% | | | -0.3% | 22.3% |  |
| Elderly >65 | 0.65% | -0.26% | 1.57% |  | | 9.1% | | | -2.9% | 22.6% |  |
| Primary care MD visits (PCMD, lag 0-2) | | |  |  | |  | | |  |  |  |
| All | **1.37%** | **0.69%** | **2.06%** |  | | **15.7%** | | | **7.1%** | **25.0%** |  |
| Children 0 – 17 | **1.07%** | **0.02%** | **2.14%** |  | | 9.8% | | | -2.6% | 23.9% |  |
| Adults 18 – 65 | **1.39%** | **0.65%** | **2.14%** |  | | **18.0%** | | | **8.6%** | **28.3%** |  |
| Elderly >65 | **1.69%** | **0.46%** | **2.94%** |  | | 14.9% | | | -0.5% | 32.6% |  |
| **Years 2012-14** |  |  |  |  | |  | | |  |  |  |
| Asthma medication (AMD, lag 0 – 2)* | | |  |  | |  | | |  |  |  |
| All | **1.24%** | **0.47%** | **2.01%** |  | | **12.0%** | | | **1.9%** | **23.1%** |  |
| Children 0 – 17 | 0.65% | -0.40% | 1.72% |  | | **15.1%** | | | **0.9%** | **31.4%** |  |
| Adults 18 – 65 | **1.27%** | **0.40%** | **2.14%** |  | | **12.0%** | | | **0.7%** | **24.7%** |  |
| Elderly >65 | **1.66%** | **0.67%** | **2.66%** |  | | 11.8% | | | -1.2% | 26.4% |  |
| Primary care MD visits (PCMD, lag 0-2) | | |  |  | |  | | |  |  |  |
| All | **1.23%** | **0.63%** | **1.82%** |  | | **17.5%** | | | **8.8%** | **26.9%** |  |
| Children 0 – 17 | 0.54% | -0.36% | 1.44% |  | | 5.9% | | | -5.7% | 18.9% |  |
| Adults 18 – 65 | **1.47%** | **0.84%** | **2.11%** |  | | **24.2%** | | | **14.3%** | **34.8%** |  |
| Elderly >65 | **1.28%** | **0.24%** | **2.33%** |  | | 14.6% | | | -0.9% | 32.5% |  |
| Hospital emergency department (HED, lag 0-2) | | | | | | | | |  |  |  |
| All | 0.31% | -0.69% | 1.32% |  | | 2.4% | | | -10.0% | 16.6% |  |
| Children 0-17 | -0.75% | -2.78% | 1.32% |  | | -11.7% | | | -31.9% | 14.4% |  |
| Adults 18-65 | -0.08% | -1.54% | 1.40% |  | | 4.8% | | | -13.6% | 27.0% |  |
| Elderly >65 | **1.60%** | **0.06%** | **3.16%** |  | | 13.2% | | | -8.4% | 39.9% |  |

Models are adjusted for NO_2_, PM_10_, and relative humidity at the same lags as SO_2_, odd holidays, day of week, the outcome at lag1, and with splines for temperature, season, and overall time trend (k=5 was set for AMD). n=1544 and 1017 for the analysis of the years 2012-14.

# Supplementary figures

## Figure S1 Time series of daily exposure and outcomes 2010-2014.


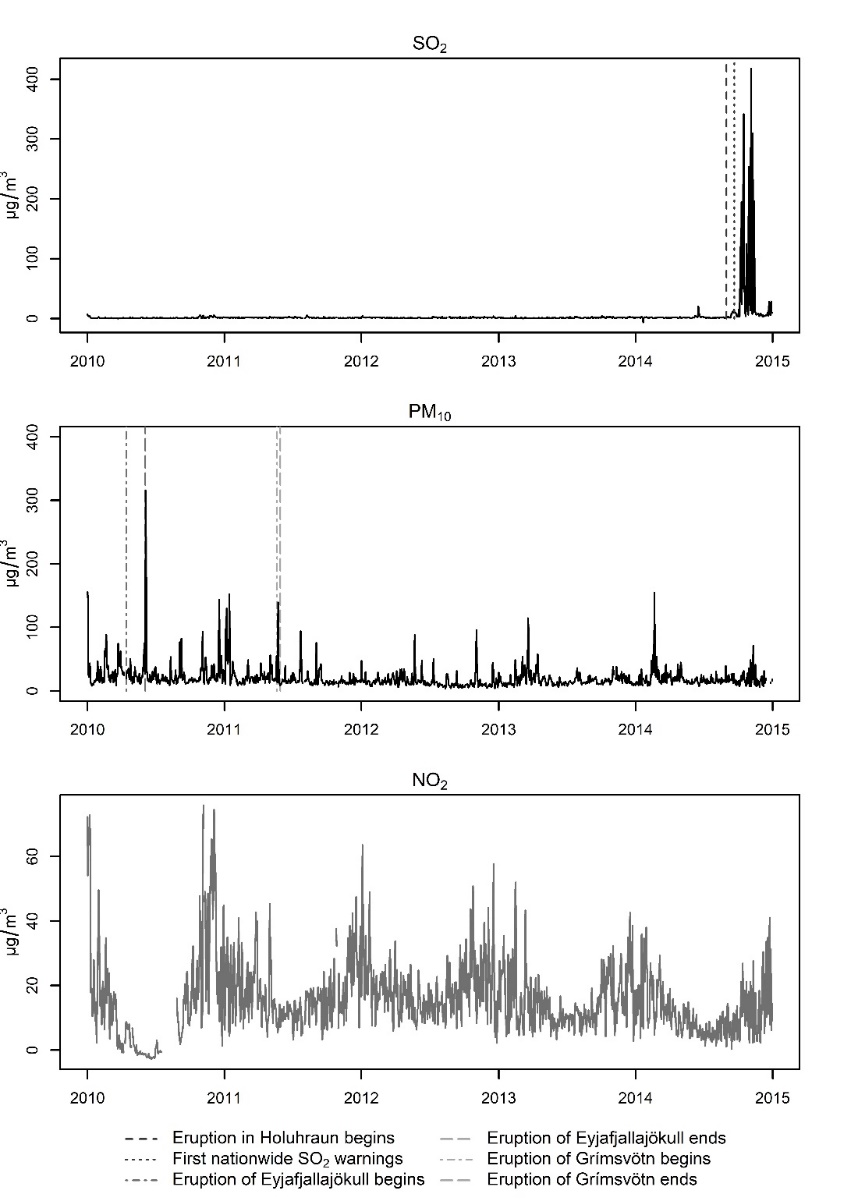


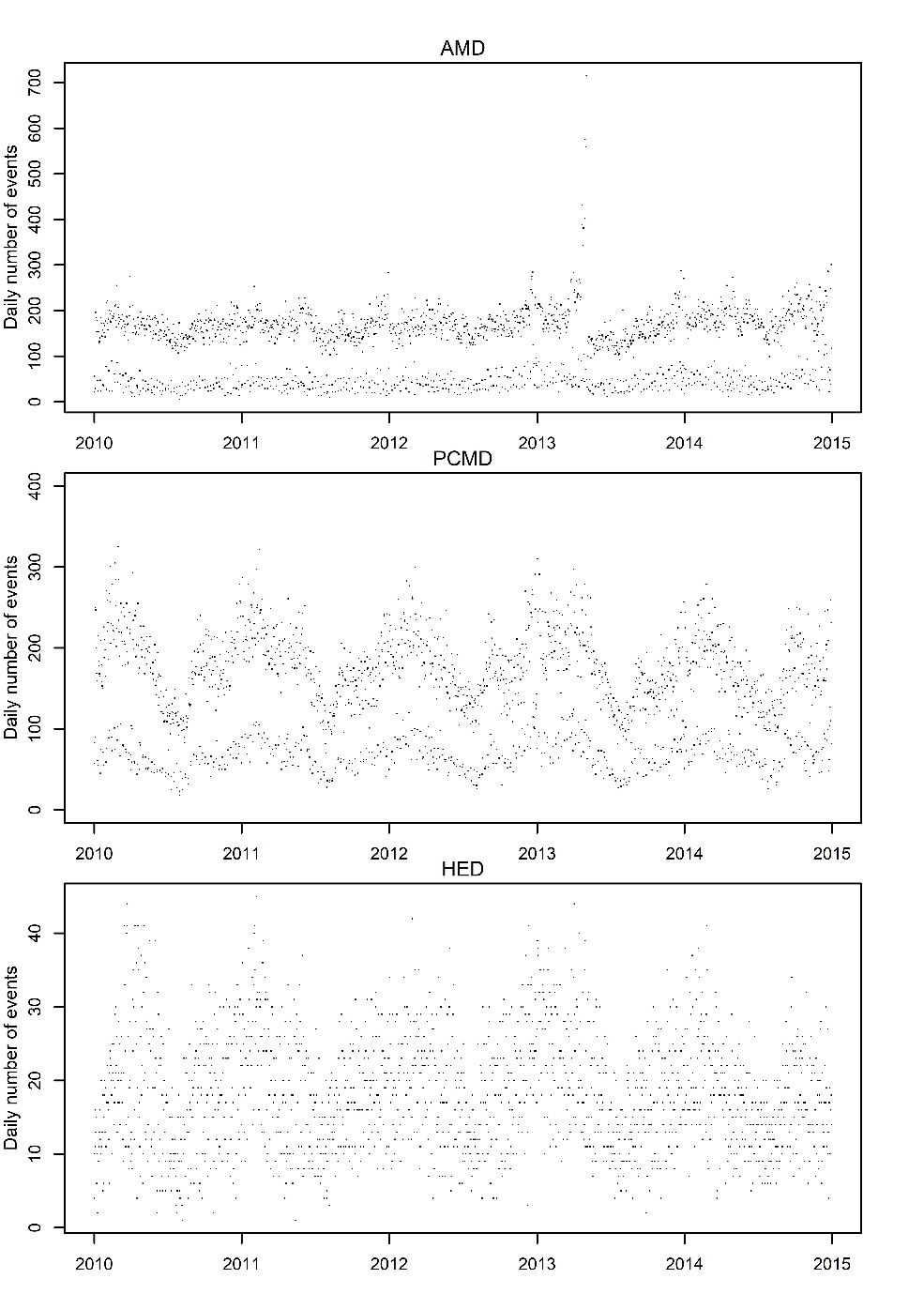


## Figure S2 a-d High SO_2_ (>125 ug/m^3^) days and associations with respiratory health outcomes across lags 0-10 (plotted using DLNM^[[1]](#footnote-2)^)


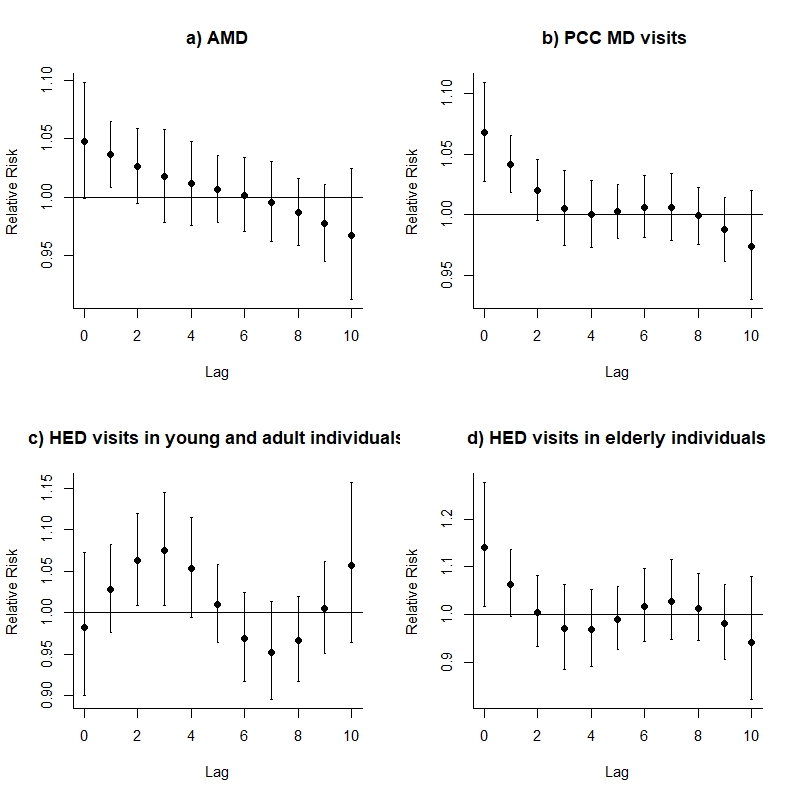


## Figure S3 a-c Concentration-response for SO_2_ and health outcomes (plotted using DLNM^[[2]](#footnote-3)^)


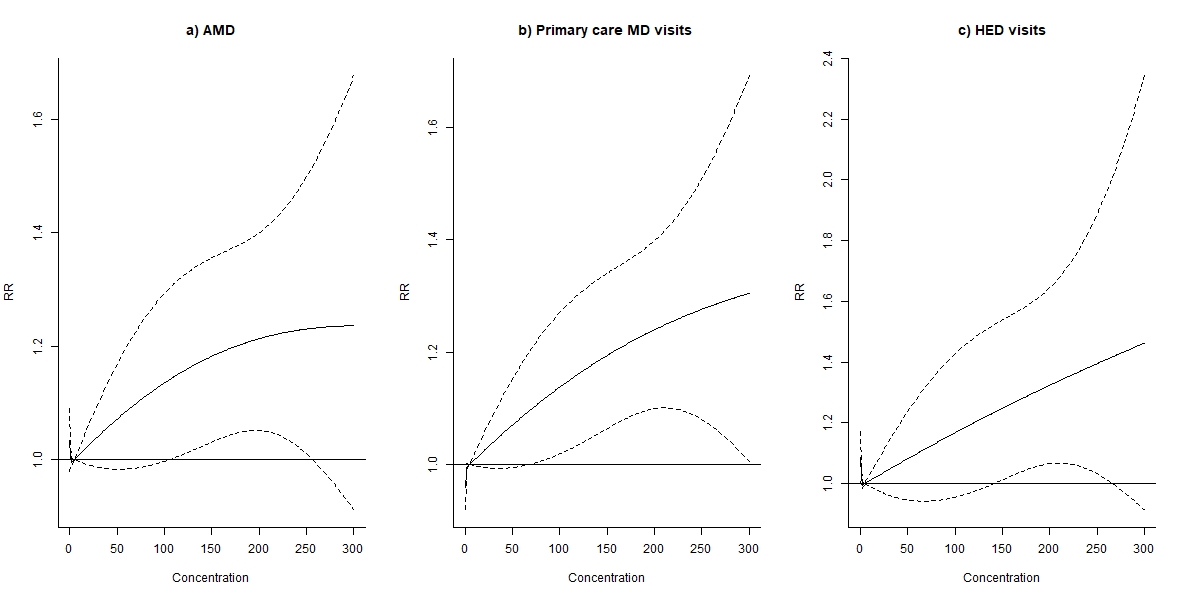


## Figure S4 a-d Associations between high SO_2_ days (>125 ug/m^3^) and subcategories of PCMD and HED (plotted using DLNM)^[[3]](#footnote-4)^

##
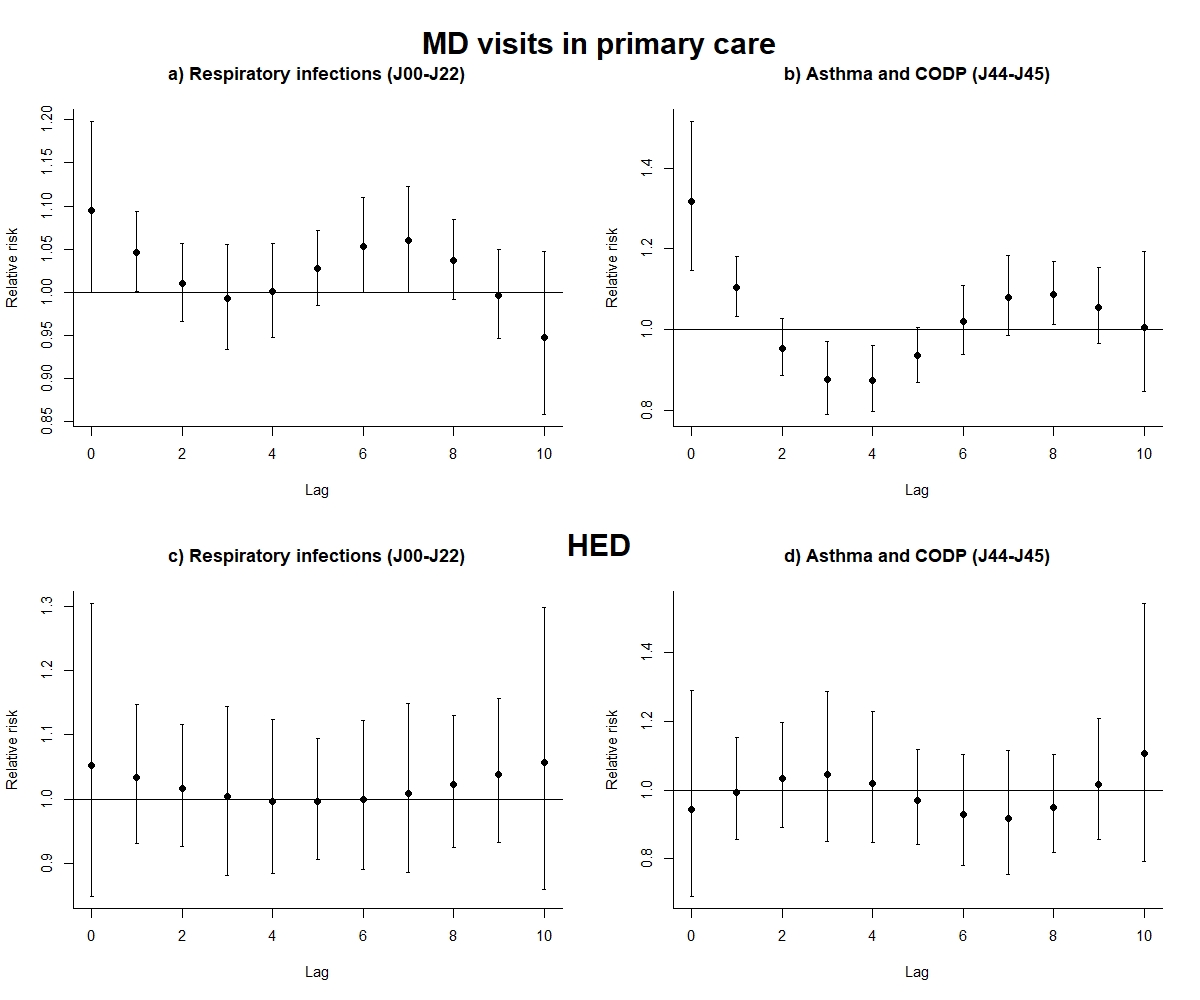
 Figure S5 a-d Lag-associations to lag 20 (plotted using DLNM^[[4]](#footnote-5)^)


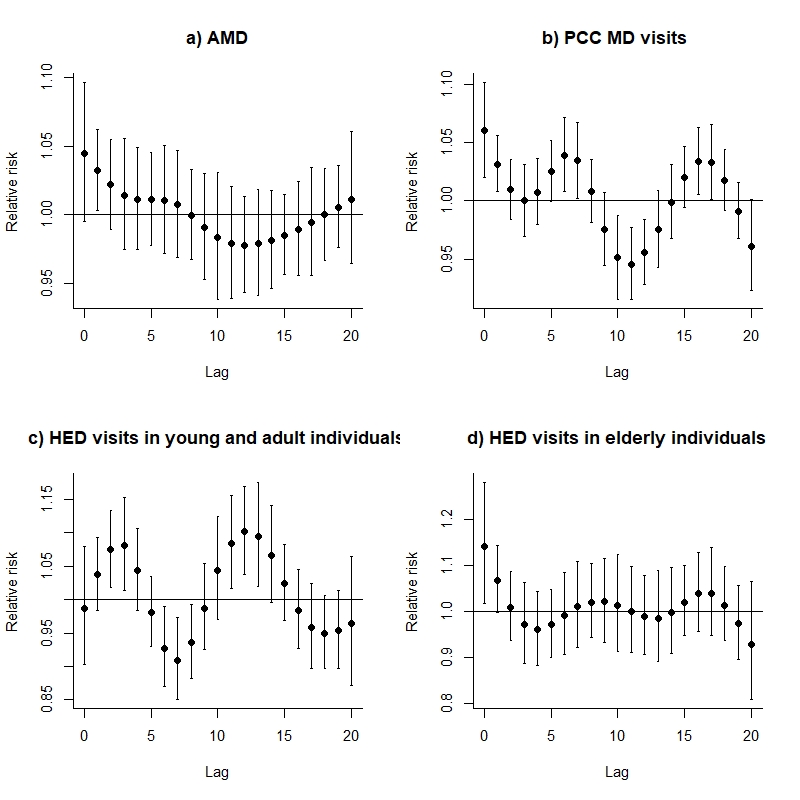


# Data selection flow diagrams

Flow diagrams of data selection of register data extracted for the study period 2010-01-01 to 2014-12-31.

First the data was selected based on the data set of events (instances, diagnoses, or, in data terms, lines), then the number of unique individuals in the data were identified* for extracting demographic information for the description of the study population characteristics found in Table 1.The time series used in Table 2 and further analysis was aggregated from the events based data.**

## Flow diagram 1: Anti-asthma medication dispensing (AMD data)

|  | **In data** |  | **Excluded** | **Cause** |
| --- | --- | --- | --- | --- |
| **Total events:** | 436 579 |  |  |  |
|  |  |  | 13 419 | No valid id |
| **Valid ID:** | 423 160 |  |  |  |
|  |  |  |  | (3803 No post code) |
|  |  |  | 3856 | (2602 No age) |
|  |  |  |  | (2643 No sex) |
| **Not missing data:** | 419 304 |  |  |  |
|  |  |  | 158 835 | Lives outside capital region |
| **Residence in capital area:** | **260 469** |  |  |  |
| Aggregate to unique individuals* | | | Aggregate to time series** | |
| **Individuals:** | **48 014** |  | **1826 days** | |

## Flow diagram 2: Primary care center MD visits (PCMD data) for respiratory diagnoses

|  | **In data** |  | **Excluded** | **Cause** |
| --- | --- | --- | --- | --- |
| **Total events:** | 1 427 011 |  |  | Missing demographic info:  Age, n=3,  Not in the national population register, n=3311,  No Icelandic postcode, n=21463 |
|  |  |  | 21 507 |  |
| **Not missing data:** | 1 405 504 |  |  |  |
|  |  |  | 568 001 | Lives outside capital region |
| **Residence in capital area:** | 837 503 |  |  |  |
|  |  |  | 383 955 | Other, non-respiratory diagnoses |
| **Respiratory diagnoses** | 453 548 |  |  |  |
|  |  |  | 102 439 | Excluding visits from patients returning within 14 days for same diagnosis type |
| **Non-recurring** | 351 109 |  |  |  |
|  |  |  | 91 055 | Telephone consultations or “other” |
| **MD consultations** | 260 054 |  |  |  |
| Aggregate to unique individuals* | | | Aggregate to time series** | |
|  | **110 806** |  | **1826 days** | |

## Flow diagram 3: Hospital emergency department visits (HED data) for respiratory diagnoses

|  | **In data** |  | **Excluded** | **Cause** | |
| --- | --- | --- | --- | --- | --- |
| **Total events:** | 361 512 |  |  |  | |
|  |  |  | 0 | No valid id | |
|  | 361 512 |  |  |  | |
|  |  |  | 14 370 | No postcode | |
| **Not missing data:** | 347 142 |  |  |  | |
|  |  |  | 211 440 | Patients returning within 14 days for same diagnosis type | |
| **Non-recurring** | 135 702 |  |  |  | |
|  |  |  | 56 084 | Lives outside capital region | |
| **Residence in capital area:** | 79 618 |  |  |  | |
|  |  |  | 46 207 | Non-respiratory diagnoses | |
| **Respiratory diagnosis** | 33 411* |  |  |  | |
| Aggregate to unique individuals* | | | Aggregate to time series** | | |
|  | 20 725 |  |  | | |
| *for analysis of admissions only, 6471 admissions were selected, and 4322 individuals were found in this data. | | | | |  |

1. *Crossbasis* defined to predict for values of SO_2_ from 0 to125 µg/m^3^ with a natural spline with four degrees of freedom for the lag-structure up to lag 10. Models were adjusted for time trends as the main models (Table 3), but not for weather or other pollutants. *Crosspred* function was centered at 5 and set to predict to 125 µg/m^3^. [↑](#footnote-ref-2)
2. *Crossbasis* for variable values have four degrees of freedom, lags 0- lag 2 for AMD and PCC, lag 0- lag 4 for HED. Model is adjusted for time trend as the main model (Table 3), but not weather or other pollutants. *Crosspred* function was centered at 5 and set to predict to 300. [↑](#footnote-ref-3)
3. *Crossbasis* defined to predict for values of SO_2_ from 0 to125 µg/m^3^ with a natual spline with four degrees of freedom for the lag-structure up to lag 10. Models were adjusted for time trends as the main models (Table 3), but not for weather or other pollutants. *Crosspred* function was centered at 5 and set to predict to 125 µg/m^3^. [↑](#footnote-ref-4)
4. *Crossbasis* defined to predict for values of SO_2_ from 0 to125 µg/m^3^ with a natural spline with four degrees of freedom for the lag-structure up to lag 20. Models were adjusted for time trends as the main models (Table 3), but not for weather or other pollutants. *Crosspred* function was centered at 5 and set to predict to 125 µg/m^3^. [↑](#footnote-ref-5)
